# Nanomaterials for Advanced Photocatalytic Plastic Conversion

**DOI:** 10.3390/molecules28186502

**Published:** 2023-09-07

**Authors:** Jae Young Kim, Duck Hyun Youn

**Affiliations:** 1 Korea Research Institute of Chemical Technology, 141 Gajeong-ro, Yuseong-gu, Daejeon 34114, Republic of Korea; 2 Department of Chemical Engineering, Department of Integrative Engineering for Hydrogen Safety, Kangwon National University, Chuncheon 24341, Republic of Korea

**Keywords:** nanomaterials, photocatalysis, plastic conversion, waste plastics, microplastics

## Abstract

As the disposal of waste plastic emerges as a societal problem, photocatalytic waste plastic conversion is attracting significant attention. Ultimately, for a sustainable future, the development of an eco-friendly plastic conversion technology is essential for breaking away from the current plastic use environment. Compared to conventional methods, photocatalysis can be a more environmentally friendly option for waste plastic reprocessing because it uses sunlight as an energy source under ambient temperature and pressure. In addition to this, waste plastics can be upcycled (i.e., converted into useful chemicals or fuels) to enhance their original value via photocatalytic methods. Among various strategies for improving the efficiency of the photocatalytic method, nanomaterials have played a pivotal role in suppressing charge recombination. Hence, in recent years, attempts have been made to introduce nanomaterials/nanostructures into photocatalytic plastic conversion on the basis of advances in material-based studies using simple photocatalysts. In line with this trend, the present review examines the nanomaterials/nanostructures that have been recently developed for photocatalytic plastic conversion and discusses the direction of future development.

## 1. Introduction

Since the photocatalytic activity of titanium dioxide (TiO_2_) was first demonstrated, the photocatalytic approach has been considered as an environmentally friendly method for solving various environmental issues, and the method has been applied to several research fields, including pollutant decomposition, water splitting, and carbon dioxide conversion. In particular, photocatalytic waste plastic conversion has attracted significant attention for addressing the emerging societal problem of waste plastic disposal.

To date, plastics have been consumed without much consideration for post-processing. This resulted in a 200-fold increase in the global yield of plastics, from 2 million metric tons in 1950 to 380 million metric tons in 2015, and it is expected to increase to 500 million tons by 2050 [1]. Plastic in its natural state takes hundreds of years to decompose, and has countless negative effects on the environment during this process [2,3,4,5], including the formation of fragments such as microplastics (defined as ≤5 mm). These become widely distributed in the sea and even in drinking water [6,7,8]. Microplastic travels through the food chain and contaminates marine life, animals, and humans, thereby threatening human health and the ecosystem alike [9,10,11,12,13,14]. Hence, for a sustainable future, the development of eco-friendly plastic conversion technology is essential for breaking away from the current plastic use environment.

**Figure 1:**
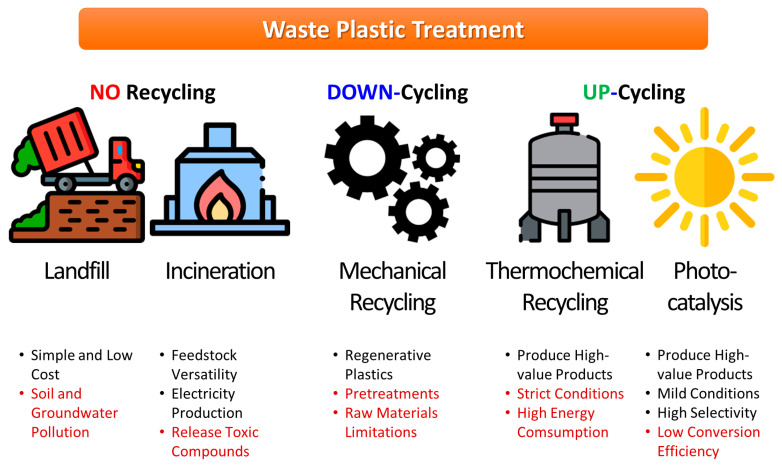
Strategies for waste plastic treatment.

Numerous technologies have been developed for reprocessing of waste plastic, including landfill, incineration, mechanical recycling, and thermochemical conversion [15,16,17,18,19] (Figure 1). Among these, incineration and landfill are the most common ways to easily dispose of waste plastic, but they have the disadvantage of emitting pollutants that are fatal to the environment, such as dioxins and greenhouse gases [20]. Alternatively, mechanical recycling is widely used for the reprocessing of waste plastic [19]. This consists of physical processes such as grinding, extruding, compounding, and pelletizing, and generally downgrades the original properties of the plastics. In addition, only uncontaminated and single-component plastics can be used as substrates for mechanical recycling. By contrast, catalytic methods for waste plastic reprocessing, including thermochemical recycling and photo/electrocatalytic methods, allow the waste plastic to be upcycled (i.e., converted into useful chemicals or fuels), thereby increasing their original value [21,22,23,24,25,26,27]. However, while thermochemical recycling generally requires high temperature and pressure, photo/electrocatalysis uses sunlight as an energy source under ambient temperature and pressure [28,29,30,31,32]. Moreover, the mild reaction conditions of photo/electrocatalysis have great potential for the precise activation of specific chemical bonds while preserving other functional groups, which can provide high selectivity toward targeted products [33]. Following C–C and C–H bond cleavages, research on C=C bond cleavage is also actively underway for the purposes of polymer degradation [34,35]. Hence, catalytic waste plastic conversion is regarded as a more forward-looking technology than mechanical recycling, and photo/electrocatalysis is regarded as the more environmentally friendly option overall.

**Figure 2:**
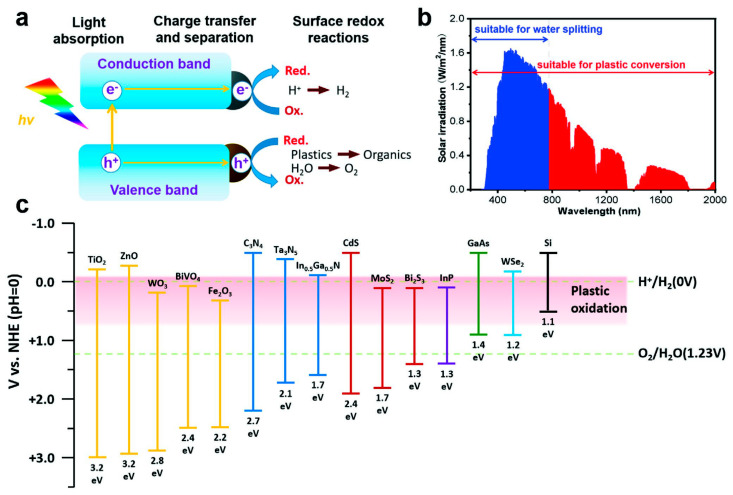
(**a**) Schematic diagram of the three main process steps in photocatalysis. (**b**) The global standard solar spectrum (AM 1.5G) and the portions of sunlight suitable for water splitting and plastic oxidation. (**c**) Bandgaps and band edge positions of various semiconductors on a potential scale versus the normal hydrogen electrode (NHE). Reproduced with permission of [33]. Copyright 2022, John Wiley and Sons.

During the photocatalytic process, plastics undergo decomposition through the absorption of energy from sunlight, generating active free radicals. When photon energy illuminates the catalysts, photogenerated electrons (e^−^) and holes (h^+^) are produced and subsequently transferred to the catalyst surface to initiate various reactions. In general, photoexcited holes can directly oxidize plastics, while other free radicals such as hydroxyl and superoxide radicals, resulting from the reaction of electrons and holes with O_2_ or H_2_O, can also contribute to the degradation of plastics [33]. A major application of photocatalysis over the past decades has been the splitting of water into molecular oxygen and hydrogen. The potential energy required to drive the overall water splitting reaction is at least 1.6 eV, which is greater than the theoretical thermodynamic requirement (1.23 eV); this is due to the kinetic overpotentials from the hydrogen evolution reaction (HER) and the even more sluggish oxygen evolution reaction (OER) [33,36,37,38,39,40,41,42] (Figure 2). In contrast to water oxidation, however, plastic oxidation occurs much more readily due to its lower potential, which reduces the overall potential requirement for coupling with H_2_ evolution. Therefore, a wide range of photocatalysts with narrow bandgaps and low valance band positions can be used for the oxidation of plastics. In fact, plastics act as sacrificial reagents that can be oxidized by low energy photogenerated holes (e.g., methanol, lactic acid, triethanolamine, etc.) [33]. Thus, waste plastics are abundant and readily available feedstock for the generation of H_2_ or other valuable chemicals, and photocatalytic plastic conversion is a much less energy consuming process than water splitting. Therefore, a significant amount of progress is expected in the use of photocatalysis as a reprocessing method for waste plastics.

The conversion method is divided into the following categories: (i) photo-degradation, (ii) photo-reforming, and (iii) photo-conversion. In the photo-degradation process, the plastic is degraded or mineralized into CO_2_ under aerobic conditions, thus leading to nonselective oxidation reactions. By contrast, plastic waste can be selectively converted into value-added products via photo-reforming and photo-conversion. In the latter processes, anoxic conditions are usually applied, and the photo-generated holes are used to drive plastic transformations, while the remaining electrons produce H_2_ when coupled with proton reduction (photo-reforming) or other valuable products when coupled with desirable reactions such as CO_2_ reduction, etc. (photo-conversion). In terms of the type of plastic, polyethylene (PE), polypropylene (PP), polystyrene (PS), and poly vinyl chloride (PVC) are commonly investigated for photo-degradation due to their chemically inert C–C bonds. Meanwhile, polar polymers, such as polyethylene terephthalate (PET), polylactic acid (PLA), polyurethane (PUR), etc., have a relative advantage in photo-conversion/reforming because they can facilitate hydrolysis and subsequent photo-reactions.

## 2. Nanomaterials for Photocatalytic Plastic Conversion

Nanomaterials have played a pivotal role in effectively and reliably suppressing charge recombination and improving the photocatalytic efficiency [43,44,45,46,47,48,49,50,51,52]. This is because the surface area of the nanostructured photocatalyst is greatly enlarged and the travel distance of the photogenerated charges is shortened, thereby increasing the probability that the desired reaction will occur without charge recombination. Historically, numerous nanomaterials have been developed for use in photocatalytic reactions, including nanostructured photocatalysts such as nanospheres, nanodots, and nanorods, along with carbon-based materials such as graphene and carbon nitride [43,44,45,46,47,48,49,50,51,52]. Hence, in recent years, attempts have been made to introduce nanomaterials/nanostructures into photocatalytic plastic conversion on the basis of advances in material-based studies using simple photocatalysts (Table 1). In line with this trend, the present review examines the nanomaterials/nanostructures that have been recently developed for photocatalytic plastic conversion, and discusses the direction of future development.

This section introduces various examples of the nanostructures that have been developed for photocatalytic plastic conversion, including metal oxide nanostructures, carbon-based nanomaterials, and some more innovative nanostructure-based systems. The role of each nanostructure will also be discussed, along with the conversion methods used, and the types of plastic converted.

### 2.1. Metal Oxide/Sulfide Nanostructures

Among the various materials of use as photocatalysts, the metal oxides are regarded as the closest to practical use due to their good photo-stability, low cost, and non-toxicity. In addition, titanium dioxide (TiO_2_) and zinc oxide (ZnO) are the most popular because their electronic band structures are suitable for photocatalytic water splitting. However, the high charge recombination rates of these materials have always been a major obstacle to improving the photocatalytic efficiency. Therefore, various nanostructures such as nanoparticles, nanorods, and nanotubes have been developed for preventing charge recombination within the metal oxide photocatalysts, and these metal oxide nanostructures have found recent application for photocatalytic plastic conversion.

**Figure 3:**
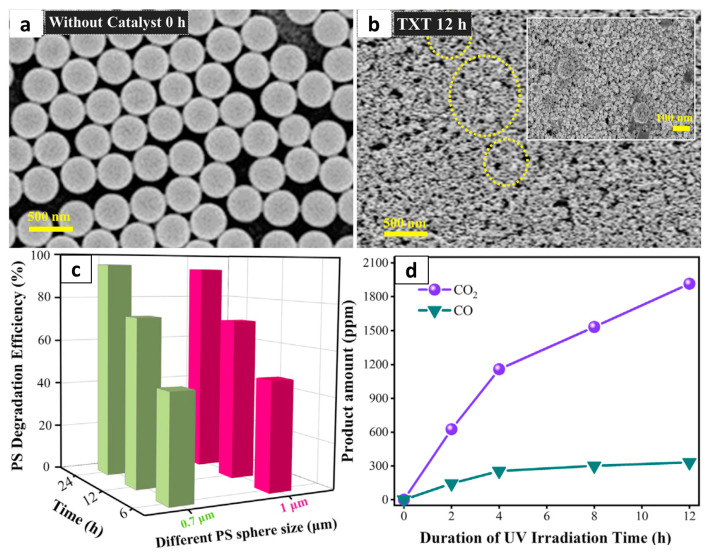
Photodegradation of PS with TiO_2_. SEM images of PS spheres on TiO_2_ after (**a**) 0 h and (**b**) 12 h irradiation under 365 nm UV light. (**c**) Degradation efficiency (%) of 1 μm and 700 nm PS on TiO_2_ film under 365 nm UV light. (**d**) Concentrations of CO_2_ and CO at different stages of a photodegradation reaction. Reproduced with permission of [53]. Copyright 2020, Elsevier.

For example, TiO_2_ nanoparticle films have been used for the photocatalytic decomposition of polyethylene (PE) and polystyrene (PS) microspheres under UV radiation (Figure 3). Thus, Zhang et al. recently reported the complete mineralization of PS microplastics by 30-nm TiO_2_ nanoparticle photocatalysts fabricated using Triton X-100 as a nonionic surfactant [53]. A decomposition of 98.4% was observed after 12 h, and complete decomposition occurred after 36 h, which is the best performance among the photocatalysts reported to date. Significant changes in the PS morphology were observed, and the 400-nm PS spheres had disappeared after 12 h of irradiation. Pure TiO_2_ nanorods were also successfully used for the degradation of microplastic particles [54]. In that study, high-density polyethylene (HDPE) microplastic was extracted from a commercially available facial scrub, and the TiO_2_ was derived from the extrapallial fluid of saltwater mussels for a more sustainable approach. At a pH of 3 and a temperature of 0 °C, the mass of microplastic particles decreased by 60% after 50 h of irradiation under a 50 W LED lamp. Meanwhile, TiO_2_ nanotubes with a large surface area were investigated for the photocatalytic degradation of PE [55]. In that study, dye sensitization was applied because other studies had demonstrated a significantly enhanced photocatalytic activity of TiO_2_ in the visible range of the solar spectrum via this approach [69,70]. The degradation rates of pure and composite PE films were measured in terms of photo-induced weight loss, and the PE films with 10% dye-sensitized TiO_2_ nanotubes showed a degradation of ~50% under visible light over a period of 45 days.

Meanwhile, ZnO stands out as a particularly promising metal oxide photocatalyst due to its band gap (3.37 eV), high redox potential, better electron mobility, and non-toxicity. In addition, ZnO is easy to synthesize and can be formed into different shapes and sizes via facile low-temperature hydrothermal growth processes [71,72]. The degradation of fragmented low-density polyethylene (LDPE) microplastic residues was demonstrated by visible light-induced heterogeneous photocatalysis activated by zinc oxide nanorods [56,73]. The results showed a 30% increase in the carbonyl index of the residues, along with an increase in brittleness accompanied by a large number of wrinkles, cracks, and cavities on the surface. The degree of oxidation was directly proportional to the catalyst surface area.

**Figure 4:**
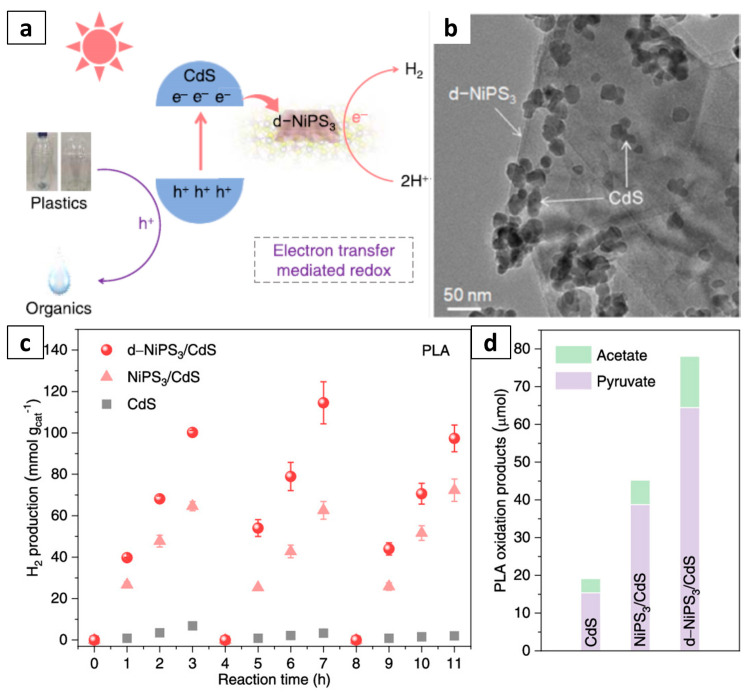
(**a**) Schematic for photo-reforming of waste plastic via defect-rich NiPS_3_ nanosheets. (**b**) HRTEM image of d-NiPS_3_/CdS. (**c**) Three (3) runs of photo-reforming for H_2_ production by CdS, NiPS_3_/CdS, and d-NiPS_3_/CdS under full-spectrum irradiation. (**d**) Organic acid products from PLA for CdS, NiPS_3_/CdS, and d-NiPS_3_/CdS following 9 h of photo-reforming. Reproduced with permission of [58]. Copyright 2023, American Chemical Society.

In addition, H_2_ and organics have been generated via the photo-reforming of a variety of widely produced plastics, including PLA, PET, and PUR, over CdS/CdO_x_ quantum dots (QDs) in alkaline solutions [57,74]. Samples of the aforementioned plastics, along with a PET water bottle, were oxidized by the photogenerated holes into organic products such as formate, acetate, and pyruvate, while the photogenerated electrons reduced protons to produce H_2_. The photoreforming of PLA yielded H_2_ with an activity of 64.3 mmol_H2_ g_cat_^−1^ h^−1^, which is one order of magnitude higher than the previous report using platinized TiO_2_ [75]. There is another work using metal sulfide materials for ambient-condition photo-reforming to convert waste plastics to hydrogen (Figure 4). Qiao et al. reported the concurrent production of H_2_ and organic chemicals from waste PLA and PET under ambient conditions via cooperative coupling of H_2_ evolution and plastic oxidation using defect-rich chalcogenide-coupled photocatalysts [58]. The defect-rich chalcogenide nanosheet-coupled photocatalysts, e.g., d-NiPS_3_/CdS, exhibits H_2_ evolution of 40 mmol g_cat_^−1^ h^−1^, 43 times that for bare CdS nanoparticles, and >100 h of stable photo-reforming of waste PET bottles and PLA cups. With the introduction of highly active d-NiPS_3_ nanosheets, spatial separation of electrons and holes was confirmed at a short timescale to promote the HER with electrons and abundant surface sites. The holes localized on photocatalysts are consumed by plastic substrates to generate organic chemicals, suppressing charge recombination, and ensuring the stability of photocatalysts.

### 2.2. Carbon-Based Nanomaterials

Carbon-based nanomaterials have attracted considerable attention in recent years for their advantages, including versatility, high stability, and large surface area. For example, graphene oxide (GO) is a layered, two-dimensional sheet structure with SP_2_ hybridized orbitals and hydrophilic oxygen functional groups located at the edges and basal planes. GO can act as an acceptor/transporter of photogenerated electrons from nanoparticles, and can reduce the recombination of photogenerated electrons and holes, thereby increasing the photocatalytic activity [60].

TiO_2_ modified with GO and silver nanoparticles has been used to degrade PE [59]. In this approach, the TiO_2_ was modified with the silver dopant by using photo-assisted deposition (PAD) and further modified with reduced graphene oxide (RGO) via ultrasonic radiation to significantly increase the catalytic performance. The as-fabricated Ag/TiO_2_/RGO catalyst exhibited a significantly high degradation efficiency of 76%, compared to 56% and 68% for pure TiO_2_ and Ag/TiO_2_, respectively. In addition, other GO-based metal oxide nanomaterials, such as GO–Cu_2_O, GO–MnO_2_, and GO–TiO_2_, have been investigated for the degradation of microplastic particles from PE in aqueous solutions [60]. These binary systems were found to be suitable for the degradation processes, and exhibited high degradation efficiencies. Thus, after 480 min, the PE particles exhibited mass losses of 48.06%, 39.54%, and 50.46% in the presence of GO–Cu_2_O, GO–MnO_2_, and GO–TiO_2_, respectively; this is compared with 35.66% in the presence of GO alone. Very recently, Dai et al. reported a biomass-derived 3D MoS_2_/RGO/cotton photocatalyst for PE photodegradation and solar-driven freshwater production [61]. This 3D nanostructure served as a versatile photothermal platform with a high evaporation rate (2.49 kg m^–2^ h^–1^), so that nearly 100% of the PE microfibers were removed from the evaporated water. Furthermore, the degradation efficiency of microplastics by MoS_2_/graphene/cotton could reach up to 32% with zero carbon emissions under a dominant contribution of O_2_^–^. This work has inspired new and advanced designs for solving the problems of removing microplastic pollutants with all-day-round evaporation.

**Figure 5:**
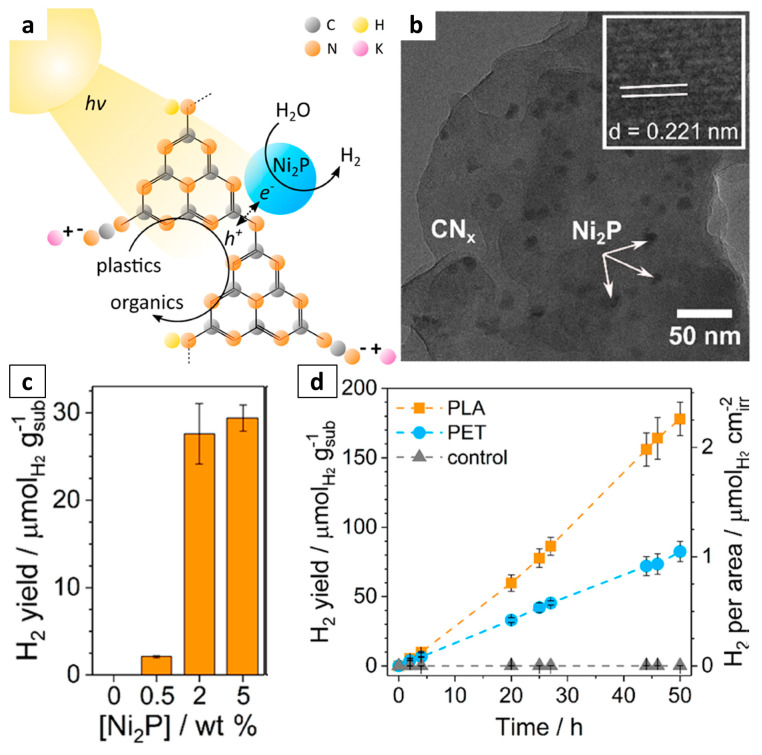
(**a**) Schematic diagram of the plastic photo-reforming process using CN*x*-Ni_2_P photocatalyst. (**b**) TEM image of CN*x*-Ni_2_P catalyst, with the inset showing the lattice spacing of Ni_2_P. (**c**) Photo-reforming of PET and PLA with CN*x*-Ni_2_P and optimization of Ni_2_P loading. (**d**) Long-term photo-reforming of PET and PLA with CN*x*-Ni_2_P. Reproduced with permission of [63]. Copyright 2019, American Chemical Society.

Polymer carbon nitride (g-C_3_N_4_) is another great example of a carbon-based nanomaterial that can provide a large surface area to the photocatalytic plastic conversion system. It has a moderate bandgap of about 2.7 eV and unique electronic properties, even exhibiting excellent photocatalytic activity under visible light [62,76]. For example, a cationic dyeable polyester modified with the g-C_3_N_4_/TiO_2_ photocatalyst was synthesized by centrifugal electrospinning and exhibited a photodegradable function under solar illumination [77]. Moreover, the cationic dyeable polyester modified with 5% g-C_3_N_4_/TiO_2_ displayed excellent self-degradation in a water environment under solar illumination. Thus, the polymer matrix was almost completely degraded after 400 h of illumination. Because the two-dimensional lamellar structure of the g-C_3_N_4_ can provide sites for the TiO_2_ to disperse evenly, the combination of these two photocatalysts can increase the light response range and reduce agglomeration of the TiO_2_. Similarly, the combination of CN*x* with a nickel phosphide cocatalyst (CN*x*-Ni_2_P) (Figure 5) has been reported for the photoreforming of plastics to H_2_ [63]. Transition electron microscopy (TEM) studies showed that the Ni_2_P nanoparticles (≈9.4 nm in diameter) were uniformly distributed across the CN*x*. During the photocatalytic process, the photogenerated electrons migrated to the Ni_2_P co-catalyst for H_2_ production, with the plastics acting as electron donors and being oxidized to organic products. The CN*x*-Ni_2_P successfully reformed PET and PLA to clean H_2_ fuel and a variety of organic chemicals under alkaline aqueous conditions.

**Figure 6:**
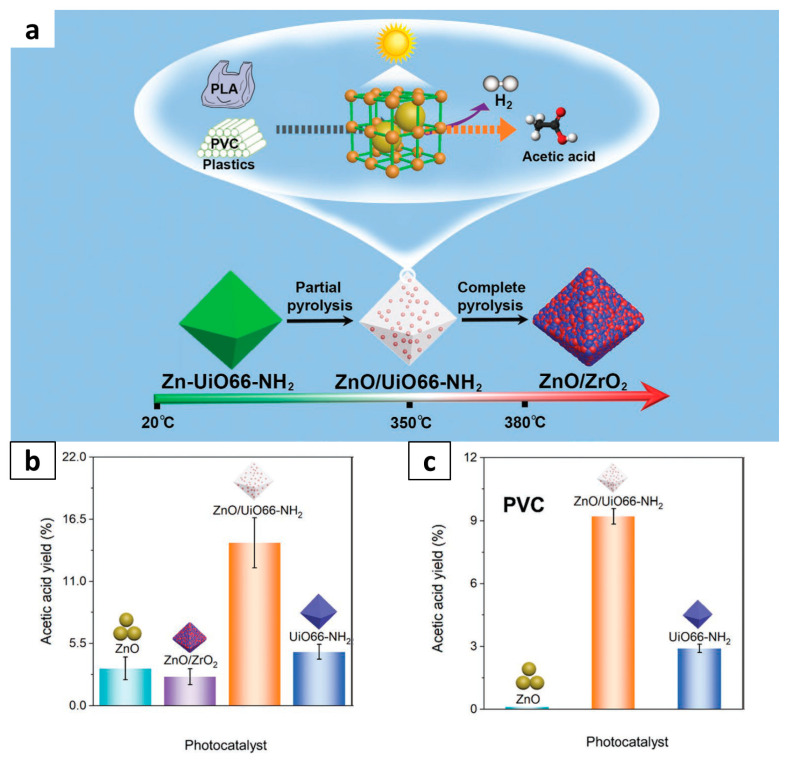
Schematic mechanism illustration for zinc oxide/metal-organic framework (ZnO/UiO66-NH_2_) heterojunction for photocatalytic valorization of waste plastics. (**a**) UiO66-NH_2_ derivatives (ZnO/UiO66-NH_2_ and ZnO/ZrO_2_) are formed by precisely controlling the calcination temperature, using Zn-UiO66-NH_2_ as a precursor. The resultant ZnO/UiO66-NH_2_ exhibits high selectivity in the photocatalytic valorization of PLA and PVC into acetic acid coupled with H_2_ production. (**b**) Acetic acid yield over ZnO, ZnO/ZrO_2_, UiO66-NH_2_, and ZnO/UiO66-NH_2_, respectively. (**c**) The acetic acid yield in PVC reaction systems. Reproduced with permission of [64]. Copyright 2023, John Wiley and Sons.

Metal-organic frameworks (MOFs), as an expanding class of porous materials, have been extensively studied [78,79]. In recent years, MOFs, including MIL-125(Ti)-NH_2_ [80], MIL-101(Fe)-NH_2_ [81], and UiO66-NH_2_ [82], have been used as novel photocatalysts in the fields of organic oxidation, CO_2_ reduction, water splitting, and NOx reduction [83]. Recently, MOF was introduced for photocatalytic conversion of waste plastic into value-added chemicals (Figure 6). The heterojunction of zinc oxide (ZnO) and MOF was developed in the form of ZnO encapsulated in porous UiO66-NH2 [64]. This strategy preserves the framework structure of UiO66-NH_2_, thus enabling the formation of ZnO with ultra-small size distributed inside the skeleton. The synergistic effect of the obtained ZnO/UiO66-NH2 heterojunction facilitates providing an efficient channel for carrier/mass transfer and guarantees structural stability. As a result, ZnO/UiO66-NH_2_ exhibits high activity for converting PLA and PVC into acetic acid, coupled with H_2_ production.

As a dual function material, photocatalysts-based fibrous membranes were reported for capturing and upcycling of plastics [65]. Polyacrylonitrile (PAN) nanofiber templates enable the growth of highly arranged metal tungstates (FeCoNiCuZn)WO_4_, denoted as XWO_4_. The effect of lattice distortion in XWO_4_ modulates the band gap structure and contributes to the upshift d-band center of XWO_4_. As a result, the modified anti-bonding state facilitates the upcycling of captured polylactic acid (PLA) towards acetic acid production, with an enhanced yield rate of 38.51 mg g_cat_^−1^ h^−1^ and improved selectivity of 73%. Thus, the developed nanofiber template strategy, a tunable electronic structure, and exploration of the structure-function relationship provide insight into tailoring the structure and composition of photocatalysts for upcycling of plastic.

### 2.3. Innovative Systems Based on Nanostructures

**Figure 7:**
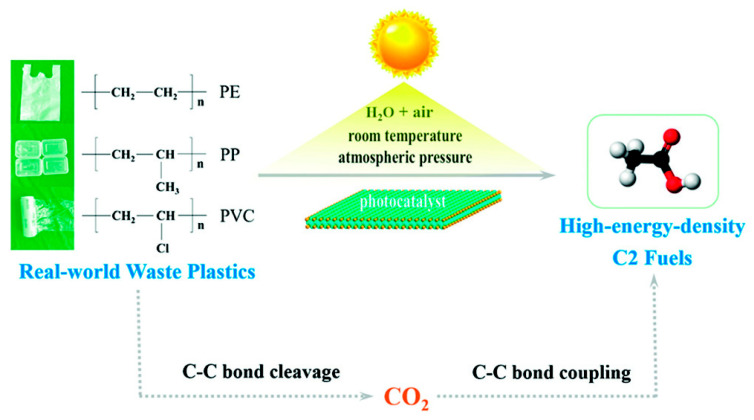
Schematic illustration for converting various waste plastics into fuels by a designed two-step pathway under ambient conditions. In other words, various plastics were degraded exclusively into CO_2_ by a photooxidative C–C bond cleavage over a photocatalyst, while the produced CO_2_ was further reduced into valuable fuels by a photoinduced C–C bond coupling over the same photocatalyst (or bifunctional catalyst). Reproduced with permission of Ref. [84]. Copyright 2020, John Wiley and Sons.

The photo-conversion of plastics is often hindered by low conversion efficiency and the excessive accumulation of CO_2_ as terminal products. To address these shortcomings, a two-step procedure has been proposed [84], involving solar-driven C–C bond cleavage and coupling of plastic photo-conversion into carbon-based fuels at normal temperature and pressure (Figure 7). This two-step pathway offers an innovative approach compared to conventional one-step photocatalytic methods, as it utilizes the relatively low-value end product, CO_2_. Nevertheless, the formation yield of the final product is limited by the modest activity of CO_2_ photoreduction over the same catalyst employed for plastic degradation. To overcome this limitation, a bifunctional catalyst should be designed, with one part efficiently degrading plastics into abundant CO_2_, and the other part dedicated to the subsequent CO_2_ photoreduction into target products with high efficiency. Furthermore, a tandem two-chamber reaction cell can be fabricated, allowing for separate plastic degradation and CO_2_ reduction without mutual interference. This setup ensures that the generated CO_2_ from the former cell is transferred into the latter one for efficient CO_2_ reduction.

Another eco-friendly approach for plastic disposal is biodegradation, where living organisms or microorganisms are capable of breaking down plastics under normal pressure and temperature [85,86,87]. These microorganisms cleave the bonds in plastics, converting them into valuable compounds. Plastic biodegradation techniques are not only economically beneficial but also environmentally friendly, as they do not produce any harmful by-products during the process [88]. Thus, biocatalysts can serve as promising bifunctional catalysts for plastic conversion. In fact, recent efforts have been made to achieve both high conversion efficiency and selectivity by combining a photo-system and a bio-system.

As an example, a biotic-abiotic photocatalytic system was designed by assembling *Methanosarcina barkeri (M. b)* and carbon dot-functionalized polymeric carbon nitride (CDPCN), by which the heat-treated biodegradable microplastic poly(lactic acid) (PLA) could be converted into CH_4_ during five successive 24-day cycles with nearly 100% CH_4_ selectivity by the assistance of additional CO_2_ [66]. CH_4_ is selected as a target product due to its high calorific value of 890 kJmol^−1^ and its compatibility with the existing energy infrastructure. As a proof of concept, the self-assembled *M. b*-CDPCN demonstrated a sustainable high-grade CH_4_ production (>99.5%), coupled with the 90.2% mineralization of PLA that was pretreated at 180 °C for 1 h. Subsequent research confirmed the real-world viability of the *M. b*-CDPCN hybrid system by effectively converting non-biodegradable microplastics such as polystyrene (PS), polyethylene terephthalate (PET), and polyurethane (PUR) films into CH_4_. This study introduces a unique and sustainable approach toward the efficient conversion of plastic wastes into valuable fuels, achieving high mineralization efficiency and product selectivity. Meanwhile, in another innovative example of the integration of photocatalysis and biocatalysis for plastic conversion, a solar-driven biocatalytic photoelectrochemical platform was reported to synthesize value-added compounds using non-recyclable real-world PET microplastics as an electron feedstock [67]. A Zr-doped hematite (α-Fe_2_O_3_) photoanode extracted electrons from hydrolyzed PET solutions obtained from post-consumer PET waste and transferred them to the bioelectrocatalytic site. Carbon fiber paper (CFP)-based cathodes then received the electrons to activate redox enzymes that could drive various organic synthetic reactions. These hybrid reactions achieved total turnover numbers of 362,000 with peroxygenase and the CFP-based cathode generated valuable chemicals (for example, formate and acetate) through the combination of photoelectrocatalysis and redox biotransformations, including oxyfunctionalization of C–H bonds, reductive amination of C=O bonds, and trans-hydrogenation of C=C bonds. In this system, the hematite nanorods and carbon fibers provided large surface areas for plastic conversion, resulting in the improved charge separation efficiencies (Δ*η*_bulk_ of 3.5–10%, Δ*η*_surface_ of 15–60%) and the decreased charge transfer resistance (by up to 17 times). Furthermore, the Zr:α-Fe_2_O_3_ photoanode reformed real-world PET items for over ten days.

There is another example using α-Fe_2_O_3_ photoanode for efficient waste plastic valorization [68]. Jiang et al. reported a photoelectrochemical (PEC) approach for valorization of waste PET to produce formate coupled with green hydrogen production. An alternative route to photocatalytic and electrocatalytic is photoelectrochemical (PEC) technology, which has emerged as a powerful tool that combines both the advantages of photocatalysis and electrocatalysis [89]. Compared with photocatalysis, the PEC system can achieve highly efficient charge separation with the assistance of applied electrical bias, and allows the separation of anodic and cathodic processes with minimized crossover reactions. In comparison with electrocatalysis, the PEC system reduces external bias and saves electricity due to the photovoltage effect, and can potentially avoid overoxidation and reduce side reactions in order to achieve better selectivity in the lower external bias region. In this work, a nickel phosphate cocatalyst on α-Fe_2_O_3_ photoelectrode (Ni-Pi/α-Fe_2_O_3_) is developed to achieve a high Faradaic efficiency to formate over 85%. Benefiting from more favorable thermodynamics of PET oxidation than that of water oxidation, the cell voltage for integrated PET oxidation-hydrogen production system is significantly reduced by ~270 mV when compared with conventional water splitting to achieve the same current density.

## 3. Conclusions and Perspectives

**Figure 8:**
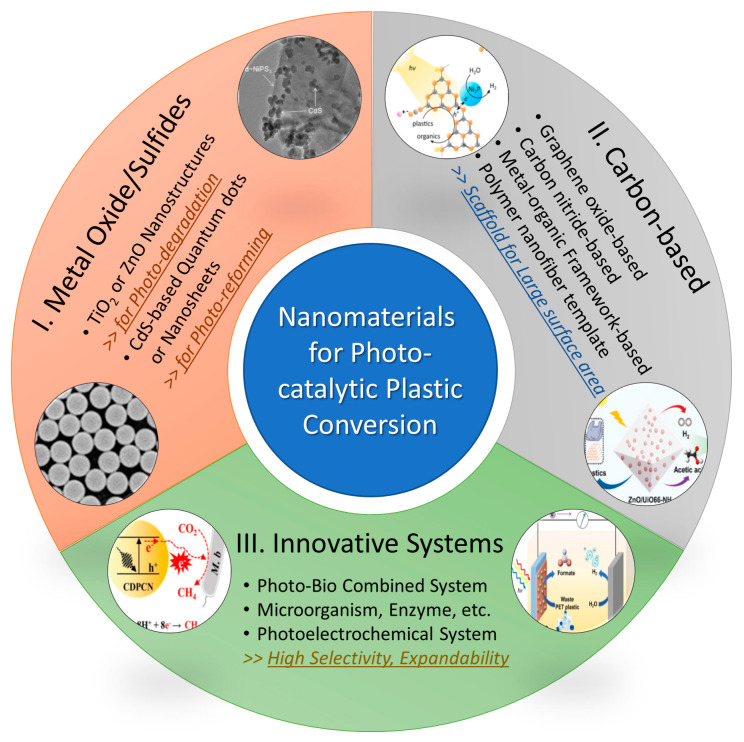
Summary of nanomaterials for photocatalytic plastic conversion. Insets reproduced with permission of Refs. [53,58,63,64,66,68]. Copyright 2020, Elsevier. Copyright 2023, American Chemical Society. Copyright 2019, American Chemical Society. Copyright 2023, John Wiley and Sons. Copyright 2022, John Wiley and Sons. Copyright 2023, Elsevier.

In the previous section, the nanomaterials for advanced photocatalytic plastic conversion were introduced under the three categories of metal oxide/sulfide nanostructures, carbon-based nanomaterials, and innovative systems based on nanostructures (Figure 8). Nanostructures have progressed steadily from single-component to multi-component and thin-film (electrode) structures. According to the literature, metal oxide nanostructures began to be used for photo-degradation of waste plastics. Recently, sulfide nanostructures and carbon-based nanomaterials seem to be expanding the application field to photo-reforming/conversion (Table 1). Among them, graphene oxides (GO) and carbon nitrides were the most popular scaffolds, providing a large surface area for photocatalytic plastic conversion [59,60,61,62,63]. In particular, a 3D GO nanostructure was also reported to be used as the platform for both removal and photo-degradation of microplastics [61]. Most recently, the examples of using MOF and nanofiber has been reported as a scaffold with a large surface area [64,65], and it is expected that more types of nanostructures will be used for photocatalytic plastic conversion in the future. Meanwhile, there have been novel attempts such as introducing a biotic system or configuring a cell with two or more film-type electrodes [66,67,68]. In the photo-bio hybrid systems, the introduction of microorganisms or enzymes can greatly help to increase the selectivity of the target product [66,67]. Furthermore, some photoelectrochemical systems have been reported using the film-type photocatalyst nanostructure as an electrode [67,68]. These systems are noteworthy for their expandability because they have diversity in material selection and system configuration. In the future, it is expected that nanomaterials/structures will continue to expand their range of applications beyond the present fields and limitations.

**Figure 9:**
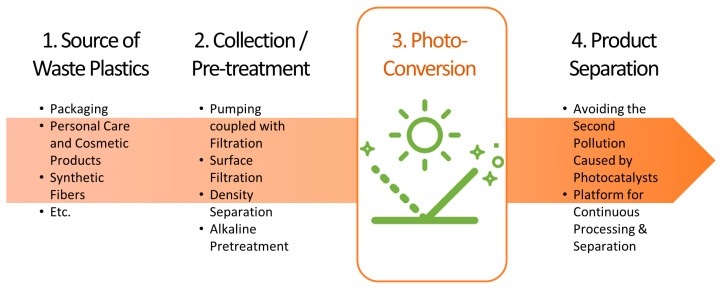
Considerations based on waste plastic lifecycle when developing photocatalytic plastic conversion system.

Meanwhile, some requirements are expected for nanomaterials from the perspective of the life cycle of waste plastics (Figure 9). First, the source of plastic waste should be considered. Plastic waste can occur in a variety of places and, in the case of wastewater, various types of microplastics are present and are difficult to separate [90]. Major sources of these microplastics include personal care and cosmetic products (PCCPs), along with synthetic fibers. Approximately 35% of the microplastics identified in the aquatic environment might be released from synthetic fibers during washing [91]. Therefore, the most suitable photocatalytic method may vary depending on the type and nature of the discharged waste plastics. Second, the collection and pre-treatment methods of the waste plastics must be considered. To date, the methods used for collecting microplastics from wastewater have included (i) pumping coupled with filtration and (ii) surface filtration. Of these, the pumping method coupled with filtration can be used for the collection of hundreds of liters of wastewater. For example, a device was developed that contains four stainless steel mesh screens with sizes of 25, 100, 190, and 500 mm, respectively [92]. Using this device, a large volume of samples could be treated continuously, and the in-situ separation of microplastics was possible based on their size. Meanwhile, surface filtration is a highly effective sampling technique for collecting thousands of cubic meters of wastewater, but is only applicable at waterfalls. After collecting the microplastics, some pre-treatment processes, such as density separation, digestion, alkaline pretreatment, etc., may be required. The extraction of microplastics from liquid is commonly achieved by density separation, which involves the mixing of a liquid of defined density with the sample [93], while digestion is required to remove biogenic materials from wastewater due to its complex composition. To date, the most effective digestion approach involves the incubation of the sample in 30% (*v*/*v*) hydrogen peroxide (H_2_O_2_) [94]. In this approach, Fenton’s reagent (a mixture of H_2_O_2_ and ferrous ion (Fe^2+^)) is commonly used to reduce the amount H_2_O_2_ [95]. Alkaline pretreatment is also commonly used in plastic photoreforming studies, where it increases the plastic solubilization and facilitates the contact between the photocatalyst and the plastic substrate [33]. As introduced in the Section 2.1, PLA, PET, and PUR have converted into H_2_ and organics over CdS/CdOx quantum dots (QDs) in alkaline solutions [57,74]. However, such a treatment is not desirable due to the cost and negative impact on the environment. Hence, the state of the plastic source used for photocatalytic conversion may vary depending on the collection and/or pre-treatment method used. It is necessary to find an optimal system according to the type, size, and presence of impurities in the plastic source. Alternatively, it would be desirable to develop a photocatalytic conversion system that can simplify the pre/post-treatment processes. However, the final consideration, and probably the most important factor in designing a photocatalytic plastic conversion system based on nanostructures, is the separation of the products after the reaction. In the case of photo-degradation, separation of the products is not particularly required, but the nanostructure used for degradation should not cause other environmental pollution. In the case of photo-reforming/conversion, the considerations are more complicated. Ideally, the nanostructure should provide a platform for continuous processing and product separation simultaneously during the reaction. This might be achieved by fabricating the nanostructure in the form of a film. Film-type nanostructures can have strengths in reuse and product separation while staying in place by themselves. In brief, when designing a photocatalytic plastic conversion system based on nanostructures, the entire process from the collection of plastics to the separation of products should be considered in order to accelerate future practical use.

## Figures and Tables

**Table 1:** Typical photocatalyst/nanostructures for plastic conversion, along with the types of plastics converted, methods used, corresponding performance results and literature references.

Photocatalyst/Nanostructure	Plastic	Method	Efficiency/Yield	Time	Ref.
TiO_2_ nanoparticle film	PS	Photo-degradation	98.4%	12 h	[53]
TiO_2_ nanorods	PE	Photo-degradation	6%	20 h	[54]
TiO_2_ nanotubes	PE	Photo-degradation	67%	15 days	[55]
ZnO nanorods	PE	Photo-degradation	30%	175 h	[56]
CdS/CdOx quantum dots	PLA	Photo-reforming	64.3 mmol_H2_ g_cat_^–1^ h^–1^	–	[57]
d-NiPS_3_/CdS nanosheets	PLA	Photo-reforming	40 mmol_H2_ g_cat_^–1^ h^–1^	–	[58]
d-NiPS_3_/CdS nanosheets	PET	Photo-reforming	32 mmol_H2_ g_cat_^–1^ h^–1^	–	[58]
RGO-Ag/TiO_2_	PE	Photo-degradation	76%	4 h	[59]
GO-Cu_2_O	PE	Photo-degradation	48.06%	8 h	[60]
GO-MnO_2_	PE	Photo-degradation	39.54%	8 h	[60]
MoS_2_/RGO/cotton	PE	Photo-degradation	32%	1 h	[61]
g-C_3_N_4_/TiO_2_	PE	Photo-degradation	99%	400 h	[62]
CN*x*-Ni_2_P	PE	Photo-reforming	111 μmol_H2_ gcat^–1^	–	[63]
CN*x*-Ni_2_P	PLA	Photo-reforming	211 μmol_H2_ gcat^–1^	–	[63]
ZnO/UiO66-NH_2_ (MOF)	PLA	Photo-conversion	14.4%, S_CH3OOH_: 91.6%	–	[64]
ZnO/UiO66-NH_2_ (MOF)	PVC	Photo-conversion	9%	–	[64]
XWO_4_/PAN nanofiber ^1^	PLA	Photo-conversion	38.51 mg g_cat_^−1^ h^−1^	–	[65]
*M. b*-CDPCN	PLA	Photo-conversion	90.2%, S_CH4_: 99.5%	1 h	[66]
Zr:Fe_2_O_3_‖carbon|enzyme	PET	Photo-conversion	TON: 362 k	–	[67]
Ni-Pi/α-Fe_2_O_3_	PET	Photo-conversion	60 μmol_formate_ cm^−2^	5 h	[68]

^1^ XWO_4_: (FeCoNiCuZn)WO_4_. PAN: polyacrylonitrile.

## Data Availability

Not applicable.

## References

[1] Geyer R., Jambeck J.R., Law K.L. (2017). Production, use, and fate of all plastics ever made. Sci. Adv..

[2] MacLeod M., Arp H.P.H., Tekman M.B., Jahnke A. (2021). The global threat from plastic pollution. Science.

[3] Cornwall W. (2021). The plastic eaters. Science.

[4] Law K.L., Thomson R.C. (2014). Microplastics in the seas. Science.

[5] Barnes D.K.A., Galgani F., Thomson R.C., Barlaz M. (2009). Accumulation and fragmentation of plastic debris in global environments. Philos. Trans. R. Soc. B.

[6] Mason S.A., Welch V.G., Neratko J. (2018). Synthetic polymer contamination in bottled water. Front. Chem..

[7] Pivokonsky M., Cermakova L., Novotna K., Peer P., Cajthaml T., Janda V. (2018). Occurrence of microplastics in raw and treated drinking water. Sci. Total Environ..

[8] Wang J., Tan Z., Peng J., Qiu Q., Li M. (2016). The behaviors of microplastics in the marine environment. Mar. Environ. Res..

[9] Santos R.G., Machovsky-Capuska G.E., Andrades R. (2021). Plastic ingestion as an evolutionary trap: Toward a holistic understanding. Science.

[10] Kang J., Zhou L., Duan X., Sun H., Ao Z., Wang S. (2019). Degradation of cosmetic microplastics via functionalized carbon nanosprings. Matter.

[11] Karbalaei S., Hanachi P., Walker T.R., Cole M. (2018). Occurrence, sources, human health impacts and mitigation of microplastic pollution. Environ. Sci. Pollut. Res. Int..

[12] Barboza L.G.A., Vethaak A.D., Lavorante B.R., Lundebye A.-K., Guilhermino L. (2018). Marine microplastic debris: An emerging issue for food security, food safety and human health. Mar. Pollut. Bull..

[13] Jambeck J.R., Geyer R., Wilcox C., Siegler T.R., Perryman M., Andrady A., Narayan R., Law K.L. (2015). Plastic waste inputs from land into the ocean. Science.

[14] Cozar A., Echevarria F., Gonzalez-Gordillo J.I., Irigoien X., Ubeda B., Hernandez-Leon S., Palma A.T., Navarro S., Garcia-de-Lomas J., Ruiz A. (2014). Plastic debris in the open ocean. Proc. Natl. Acad. Sci. USA.

[15] Kasmuri N., Tarmizi N.A.A., Mojiri A. (2022). Occurrence, impact, toxicity, and degradation methods of microplastics in environment—A review. Environ. Sci. Pollut. Res..

[16] Editorials Team (2021). Making plastics sustainable isn’t the whole solution. Nature.

[17] Chen Y., Cui Z., Cui X., Liu W., Wang X., Li X., Li S. (2019). Life cycle assessment of end-of-life treatments of waste plastics in China. Resour. Conserv. Recycl..

[18] Ragaert T., Delva L., Van Geem K. (2017). Mechanical and chemical recycling of solid plastic waste. Waste Manag..

[19] Garcia J.M., Robertson M.L. (2017). The future of plastics recycling. Science.

[20] Ashworth D.C., Elliott P., Toledano M.B. (2014). Waste incineration and adverse birth and neonatal outcomes: A systematic review. Environ. Int..

[21] Su K., Liu H., Zhang C., Wang F. (2022). Photocatalytic conversion of waste plastics to low carbon number organic products. Chin. J. Catal..

[22] Wang Y., Wu K., Liu Q., Zhang H. (2021). Low chlorine oil production through fast pyrolysis of mixed plastics combined with hydrothermal dechlorination pretreatment. Process. Saf. Environ. Prot..

[23] Huang X., Zhang K., Peng B., Wang G., Muhler M., Wang F. (2021). Ceria-based materials for thermocatalytic and photocatalytic organic synthesis. ACS Catal..

[24] Chen A., Yang M.-Q., Wang S., Qian Q. (2021). Recent advancements in photocatalytic valorization of plastic waste to chemicals and fuels. Front. Nanotechnol..

[25] Nanda S., Berruti F. (2020). Thermochemical conversion of plastic waste to fuels: A review. Environ. Chem. Lett..

[26] Lopez G., Artetxe M., Amutio M., Bilbao J., Olazar M. (2017). Thermochemical routes for the valorization of waste polyolefinic plastics to produce fuels and chemicals. A review. Renew. Sustain. Energy Rev..

[27] Zhang H., Nie J., Xiao R., Jin B., Dong C., Xiao G. (2014). Catalytic co-pyrolysis of biomass and different plastics (polyethylene, polypropylene, and polystyrene) to improve hydrocarbon yield in a fluidized-bed reactor. Energy Fuels.

[28] Sayre H.J., Tian L., Son M., Hart S.M., Liu X., Arias-Rotondo D.M., Rand B.P., Schlau-Cohen G.S., Scholes G.D. (2021). Solar fuels and feedstocks: The quest for renewable black gold. Energy Environ. Sci..

[29] Zhu S.S., Wang D.W. (2017). Photocatalysis: Basic principles, diverse forms of implementations and emerging scientific opportunities. Adv. Energy Mater..

[30] Li H., Zhou Y., Tu W., Ye J., Zou Z. (2015). State-of-the-art progress in diverse heterostructured photocatalysts toward promoting photocatalytic performance. Adv. Funct. Mater..

[31] Tong H., Ouyang S.X., Bi Y.P., Umezawa N., Oshikiri M., Ye J. (2012). Nano-photocatalytic materials: Possibilities and challenges. Adv. Mater..

[32] Chen X., Shen S., Guo L., Mao S.S. (2010). Semiconductor-based photocatlytic hydrogen generation. Chem. Rev..

[33] Chu S., Zhang B., Zhao X., Soo H.S., Wang F., Xiao R., Zhang H. (2022). Photocatalytic Conversion of Plastic Waste: From Photodegradation to Photosynthesis. Adv. Energy Mater..

[34] Chen Y., Chen C., Liu Y., Yu L. (2023). Probing the effect of nitrate anion in CAN: An additional opportunity to reduce the catalyst loading for aerobic oxidations. Chin. Chem. Lett..

[35] Chen X., Mao J., Liu C., Chen C., Cao H., Yu L. (2020). An unexpected generation of magnetically separable Se/Fe_3_O_4_ for catalytic degradation of polyene contaminants with molecular oxygen. Chin. Chem. Lett..

[36] Hu Y., Huang H., Feng J., Wang W., Guan H., Li Z., Zou Z. (2021). Material design and surface/interface engineering of photoelectrodes for solar water splitting. Sol. RRL.

[37] Chen Y., Feng X., Liu Y., Guan X., Burda C., Guo L. (2020). Metal oxide-based tandem cells for self-biased photoelectrochemical water splitting. ACS Energy Lett..

[38] Wang Z., Li C., Domen K. (2019). Recent developments in hegerogeneous photocatalysts for solar-driven overall water splitting. Chem. Soc. Rev..

[39] Ye S., Ding C., Li C. (2019). Chapter one—Artificial photosynthesis systems for catalytic water oxidation. Adv. Inorg. Chem..

[40] Hu C., Zhang L., Gong J. (2019). Recent progress made in the mechanism comprehension and design of electrocatalysts for alkaline water splitting. Energy Environ. Sci..

[41] Chu S., Li W., Yan Y., Hamann T., Shih I., Wang D., Mi Z. (2017). Roadmap on solar water splitting: Current status and future prospects. Nano Futures.

[42] Takanabe K. (2017). Photocatalytic water splitting: Quantitative approaches toward photocatalyst by design. ACS Catal..

[43] Reddy N.L., Rao V.N., Vijayakumar M., Santhosh R., Anandan S., Karthik M., Shankar M.V., Reddy K.R., Shetti N.P., Nadagouda M.N. (2019). A review on Frontiers in plasmonic nano-photocatalysts for hydrogen production. Int. J. Hydrogen Energy.

[44] Cui Y., Li M., Zhu N., Cheng Y., Lam S.S., Chen J., Gao U., Zhao J. (2022). Bi-based visible light-driven nano-photocatalyst: The design, synthesis, and its application in pollutant governance and energy development. Nanotoday.

[45] Zhang G., Sewell C.D., Zhang P., Mi H., Lin Z. (2020). Nanostructured photocatalysts for nitrogen fixation. Nano Energy.

[46] Ijaz M., Zafar M. (2020). Titanium dioxide nanostructures as efficient photocatalyst: Progress, challenges and perspective. Int. J. Energy Res..

[47] Li Y., Zhang D., Qiao W., Xiang H., Besenbacher F., Li Y., Su R. (2022). Nanostructured heterogeneous photocatlyst materials for green synthesis of valuable chemicals. Chem. Synth..

[48] Tian N., Huang H., Du X., Dong F., Zhang Y. (2019). Rational nanostructure design of graphitic carbon nitride for photocatalytic applications. J. Mater. Chem. A.

[49] Xu C., Anusuyadevi P.R., Aymonier C., Luque R., Marre S. (2019). Nanostructured materials for photocatalysis. Chem. Soc. Rev..

[50] Prasad C., Tang H., Liu Q., Bahadur I., Karlapudi S., Jiang Y. (2020). A latest overview on photocatalytic application of g-C_3_N_4_ based nanostructured materials for hydrogen production. Int. J. Hydrogen Energy.

[51] Ong W.-J., Putri L.K., Mohamed A.R. (2020). Rational design of carbon-based 2D nanostructures for enhanced photocatalytic CO_2_ reduction: A dimensionality perspective. Chem. Eur. J..

[52] Xiao M., Wang Z., Lyu M., Luo B., Wang S., Liu G., Cheng H.-M., Wang L. (2019). Hollow nanostructures for photocatalysis: Advantages and challenges. Adv. Mater..

[53] Nabi I., Bacha A.U.R., Li K., Cheng H., Wang T., Liu Y., Ajmal S., Yang Y., Feng Y., Zhang L. (2020). Complete photocatalytic mineralization of microplastic on TiO_2_ nanoparticle film. iScience.

[54] Ariza-Tarazona M.C., Villarreal-Chiu J.F., Barbieri V., Siligardi C., Cedillo-González E.I. (2019). New strategy for microplastic degradation: Green photocatalysis using a protein-based porous N-TiO_2_ semiconductor. Ceram. Int..

[55] Ali S.S., Qazi I.A., Arshad M., Khan Z., Voice T.C., Mehmood C.T. (2016). Photocatalytic degradation of low density polyethylene (LDPE) films using titania nanotubes. Environ. Nanotechnol. Monit. Manag..

[56] Tofa T.S., Ye F., Kunjali K.L., Dutta J. (2019). Visible light photocatalytic degradation of microplastic residues with zinc oxide nanorods. Environ. Chem. Lett..

[57] Uekert T., Kuehnel M.F., Wakerley D.W., Reisner E. (2018). Plastic waste as a feedstock for solar-driven H_2_ generation. Energy Environ. Sci..

[58] Zhang S., Li H., Wang L., Liu J., Liang G., Davey K., Ran J., Qiao S.-Z. (2023). Boosted photoreforming of plastic waste via defect-rich NiPS_3_ nanosheets. J. Am. Chem. Soc..

[59] Fadli M.H., Ibadurrohman M., Slamet S. (2021). Microplastic pollutant degradation in water using modified TiO_2_ photocatalyst under UV-irradiation. IOP Conf. Ser. Mater. Sci. Eng..

[60] Uogintė I., Pleskytė S., Skapas M., Stanionytė S., Lujanienė G. (2022). Degradation and optimization of microplastic in aqueous solutions with graphene oxide-based nanomaterials. Int. J. Environ. Sci. Technol..

[61] Meng X., Peng X., Xue J., Wei Y., Sun Y., Dai Y. (2021). A biomass-derived, all-day-round solar evaporation platform for harvesting clean water from microplastic pollution. J. Mater. Chem. A.

[62] Pomilla F.R., Garcia-Lopez E.I., Marcì G., Palmisano L., Parrino F. (2021). Heterogeneous photocatalytic materials for sustainable formation of high value chemicals in green solvents. Mater. Today Sustain..

[63] Uekert T., Kasap H., Reisner E. (2019). Photoreforming of nonrecyclable plastic waste over a carbon nitiride/nickel phosphide catalyst. J. Am. Chem. Soc..

[64] Qin J., Dou Y., Zhou J., Candelario V.M., Anderson H.R., Hélix-Nielsen C., Zhang W. (2023). Photocatalytic valorization of plastic waste over zinc oxide encapsulated in a metal-organic framework. Adv. Funct. Mater..

[65] Wu F., Dou Y., Zhou J., Qin J., Jiang T., Yao Y., Hélix-Nielsen C., Zhang W. (2023). High-entropy (FeCoNiCuZn)WO_4_ photocatalysts-based fibrous membrane for efficient capturing and upcycling of plastic. Chem. Eng. J..

[66] Ye J., Chen Y., Gao C., Wang C., Hu A., Dong G., Chen Z., Zhou S., Xiong Y. (2022). Sustainable conversion of microplastics to methane with ultrahigh selectivity by a biotic-abiotic hybrid photocatalytic system. Angew. Chem. Int. Ed..

[67] Kim J.H., Jang J., Hilberath T., Hollmann F., Park C.B. (2022). Photoelectrocatalytic biosynthesis fuelled by microplastics. Nat. Synth..

[68] Zhang B., Zhang H., Pan Y., Shao J., Wang X., Jiang Y., Xu X., Chu S. (2023). Photoelectrochemical conversion of plastic waste into high-value chemicals coupling hydrogen production. Chem. Eng. J..

[69] Chowdhury P., Moreira J., Gomaa H., Ray A.K. (2012). Visible-solar-light-driven photocatalytic degradation of phenol with dye-sensitized TiO_2_: Parametric and kinetic study. Ind. Eng. Chem. Res..

[70] Vinu R., Polisetti S., Madras G. (2010). Dye sensitized visible light degradation of phenolic compounds. Chem. Eng. J..

[71] Qi K., Cheng B., Yu J. (2017). Review on the improvement of the photocatalytic and antibacterial activities of ZnO. J. Alloys Compd..

[72] Çolak H., Karaköse E., Duman F. (2017). High optoelectronic and anti-microbial performances of green synthesized ZnO nanoparticles using Aesculus hippocastanum. Environ. Chem. Lett..

[73] Tofa T.S., Ye F., Kunjali K.L., Dutta J. (2019). Enhanced visible light photodegradation of microplastic fragments with plasmonic platinum/zinc oxide nanorod photocatalysts. Catalysts.

[74] Wakerley D.W., Kuehnel M.F., Orchard K.L., Ly K.H., Rosser T.E., Reisner E. (2017). Solar-driven reforming of lignocellulose to H_2_ with a CdS/CdO*_x_* photocatalyst. Nat. Energy.

[75] Kawai T., Sakata T. (1981). Photocatalytic hydrogen production from water by the decomposition of poly-vinylchloride, protein, algae, dead insects, and excrement. Chem. Lett..

[76] Ong W.J., Tan L.L., Ng Y.H., Yong S.T., Chai S.P. (2016). Graphitic carbon nitride (g-C_3_N_4_)-based photocatalysts for artificial photosynthesis and environmental remediation: Are we a step closer to achieving sustainability?. Chem. Rev..

[77] Zhong Y., Zhang B., Zhu Z., Wang G., Mei X., Fang Y., Lu W. (2023). Photocatalytic-driven self-degradation of polyester microplastics under solar light. J. Polym. Environ..

[78] Yao Y., Ma Z., Dou Y., Lim S.Y., Zou J., Stamate E., Jensen J.O., Zhang W. (2022). Random occupation of multimetal sites in transition metal-organic frameworks for boosting the oxygen evolution reaction. Chem.—Eur. J..

[79] Dou Y., Zhang W., Kaiser A. (2020). Electrospinning of metal-organic frameworks for energy and environmental applications. Adv. Sci..

[80] Cheng X.M., Dao X.Y., Wang S.Q., Zhao J., Sun W.Y. (2021). Enhanced photocatalytic CO_2_ reduction activity over NH_2_-MIL-125(Ti) by facet regulation. ACS Catal..

[81] Shi H., Li C., Wang L., Wang W., Meng X. (2022). Selective reduction of nitrate into N_2_ by novel z-scheme NH_2_-MIL-101(Fe)/BiVO_4_ heterojunction with enhanced photocatalytic activity. J. Hazard. Mater..

[82] Su Y., Zhang Z., Liu H., Wang Y. (2017). Cd_0.2_Zn_0.8_S@UiO-66-NH_2_ nanocomposites as efficient and stable visible-light-driven photocatalyst for H_2_ evolution and CO_2_ reduction. Appl. Catal. B.

[83] Younis S.A., Kwon E.E., Qasim M., Kim K.H., Kim T., Kukkar D., Dou X., Ali I. (2020). Metal-organic framework as a photocatalyst: Progress in modulation strategies and environmental/energy applications. Prog. Energy Combust. Sci..

[84] Jiao X., Zheng K., Chen Q., Li X., Li Y., Shao W., Xu J., Zhu J., Pan Y., Sun Y. (2020). Photocatalytic conversion of waste plastics into C_2_ fuels under simulated natural environment conditions. Angew. Chem. Int. Ed..

[85] Shah A.A., Hasan F., Hameed A., Ahmed S. (2008). Biological degradation of plastics: A comprehensive review. Biotechnol. Adv..

[86] DelRe C., Jiang Y., Kang P., Kwon J., Hall A., Jayapurna I., Ruan Z., Ma L., Zolkin K., Li T. (2021). Near-complete depolymerization of polyesters with nano-dispersed enzymes. Nature.

[87] Tournier V., Topham C.M., Gilles A., David B., Folgoas C., Moya-Leclair E., Kamionka E., Desrousseaux M.L., Texier H., Gavalda S. (2020). An engineered PET depolymerase to break down and recycle plastic bottles. Nature.

[88] Zhang F., Zhao Y., Wang D., Yan M., Zhang J., Zhang P., Ding T., Chen L., Chen C. (2021). Current technologies for plastic waste treatment: A review. J. Clean. Prod..

[89] Wang B., Biesold G.M., Zhang M., Lin Z. (2021). Amorphous inorganic semiconductors for the development of solar cell, photoelectrocatalytic and photocatalytic applications. Chem. Soc. Rev..

[90] Hu Y., Gong M., Wang J., Bassi A. (2019). Current research trends on microplastic pollution from wastewater systems: A critical review. Rev. Environ. Sci. Biotechnol..

[91] Prata J.C. (2018). Microplastics in wastewater: State of the knowledge on sources, fate and solutions. Mar. Pollut. Bul..

[92] Ziajahromi S., Neale P.A., Rintoul L., Leusch F.D.L. (2017). Wastewater treatment plants as a pathway for microplastics: Development of a new approach to sample waterwater-based microplastics. Water. Res..

[93] Li J., Liu H., Paul Chen J. (2018). Microplastics in freshwater systems: A review on occurrence, environmental effects, and methods for microplastics detection. Water. Res..

[94] Stock F., Kochleus C., Bänsch-Baltruschat B., Brennholt N., Reifferscheid G. (2019). Sampling techniques and preparation methods for microplastic analyses in the aquatic environment—A review. Trends Anal. Chem..

[95] Tagg A.S., Harrison J.P., Ju-Nam Y., Sapp M., Bradley E.L., Sinclair C.J., Ojeda J.J. (2017). Fenton’s reagent for the rapid and efficient isolation of microplastics from wastewater. Chem. Commun..

